# Case Report: Delayed recurrence of staphylococcal scalded skin syndrome in an extremely low birth weight infant

**DOI:** 10.3389/fped.2025.1564633

**Published:** 2025-03-17

**Authors:** Sara M. Hooper, Camille M. Fung, Carrie Torr, Shelley M. Lawrence

**Affiliations:** ^1^ Primary Children’s Hospital, Intermountain Healthcare, Salt Lake City, UT, United States; ^2^ Department of Pediatrics, Division of Neonatology, Spencer Fox Eccles School of Medicine, University of Utah, Salt Lake City, UT, United States

**Keywords:** methicillin-susceptible *S. aureus*, staphylococcal scalded skin syndrome (SSSS), neonate, endotoxin ETA and ETB, desmoglein

## Abstract

Preterm infants have well-documented deficiencies in their innate and adaptive immune responses, which are indirectly correlated with their gestational age at birth. They also exhibit low levels of circulating immunoglobulins due to the lack of maternal transplacental IgG transfer during the third trimester of pregnancy. These factors place them at a particularly high risk for infectious diseases after birth. Diagnosing infections that primarily manifest through abnormal skin findings can be challenging, given overlapping characteristics attributed to bacterial and yeast pathogens. The case presented involves an infant born extremely premature with staphylococcal scalded skin syndrome (SSSS), a diagnosis rarely made in neonatal patients. However, he was initially treated for a yeast infection of his neck and skin folds, which is very common. This patient's course was complicated by a family history concerning for an undiagnosed, inherited immune deficiency. This case highlights the clinical findings and management of SSSS in preterm infants. It also details the importance of establishing a specialized multidisciplinary team to coordinate and manage the care of these patients.

## Introduction

Staphylococcal scalded skin syndrome (SSSS), also known as Ritter’s disease, is a rare condition in the neonatal intensive care unit caused by the release of serine protease exfoliative toxins by *Staphylococcus aureus* bacterial strains (1, 2). In the United States, the mean annual incidence of SSSS is estimated to be 7.67 (range, 1.83–11.88) per million children but significantly increases for those <2 years of age at 45.1 cases per million infants. SSSS is caused by the hematogenous dissemination of staphylococcal exfoliative toxins (ET) A or B (1, 2). Older children and adults have less susceptibility to this condition because of the accumulation of sufficient levels of anti-ETA and anti-ETB antibodies. Infants, especially those born preterm, can have low levels of anti-ET antibodies due to lack of transplacental transfer, which occurs during the third trimester of pregnancy. Alternatively, young infants and children may experience low circulating antibody levels from lack of exposure to the antigen. Therefore, SSSS mainly affects infants <1 year of age (cumulative incidence of 7.5 per 100,000 population), and cases have been rising over the last few decades, with an increased incidence of 47.1% between 2010 and 2014. However, nearly 80% of these cases have occurred in infants <1 year of age (3–5). This case details the presentation of an extremely low birth weight infant who developed SSSS at about 6 weeks of life, with recurrence 2 months later, and the clinical management of this condition.

## Case presentation

This case concerns a 680 g male infant, born at 23 weeks and 6 days via classical Cesarean section following preterm rupture of membranes (11 h before delivery) of clear fluid and preterm labor. The G7P6 mother presented to the labor and delivery department 5 days before this baby's birth due to uterine contractions. The mother received indomethacin, nifedipine, magnesium sulfate, and a complete antenatal course of betamethasone prior to delivery. After membrane rupture, the mother also received azithromycin and cefazolin >4 h prior to delivery. The mother's history is significant for a previous loss of a neonate due to late-onset group B *Streptococcus* (GBS) sepsis. Apgar scores were 3, 6, and 7 at 1, 5, and 10 min, respectively. The baby was intubated and received surfactant after birth. He received a total of three doses of Curosurf after birth, with the second and third doses administered on the second day of life. Maternal labs reported that she was blood type AB positive, antibody screen negative, rubella immune, and recent GBS vaginal/rectal culture negative. The maternal infectious disease panel was negative for syphilis (RPR negative), hepatitis B negative, and HIV negative. The baby received empiric ampicillin and gentamicin for 48 h, while early-onset sepsis was ruled out.

At 3 weeks of age, the neonate was transferred from a community neonatal intensive care (NICU) to Primary Children's Hospital in Salt Lake City due to concerns for possible bowel obstruction for which vancomycin and piperacillin/tazobactam were initiated. Although this condition was later ruled out, a diagnosis of stage I necrotizing enterocolitis was documented, and he received a 7-day course of piperacillin/tazobactam. He also received treatment for omphalitis and suspected yeast infection on his neck and axilla at this time, for which he received vancomycin for 72 h and nystatin powder topically to affected regions until it was resolved. His course was also complicated by bilateral grade II intraventricular hemorrhage and esophageal perforation, which resolved without the need for surgical intervention. He also had closure of his patent ductus arteriosus with a Piccolo Occluder at approximately 4 weeks of life.

**Figure 1:**
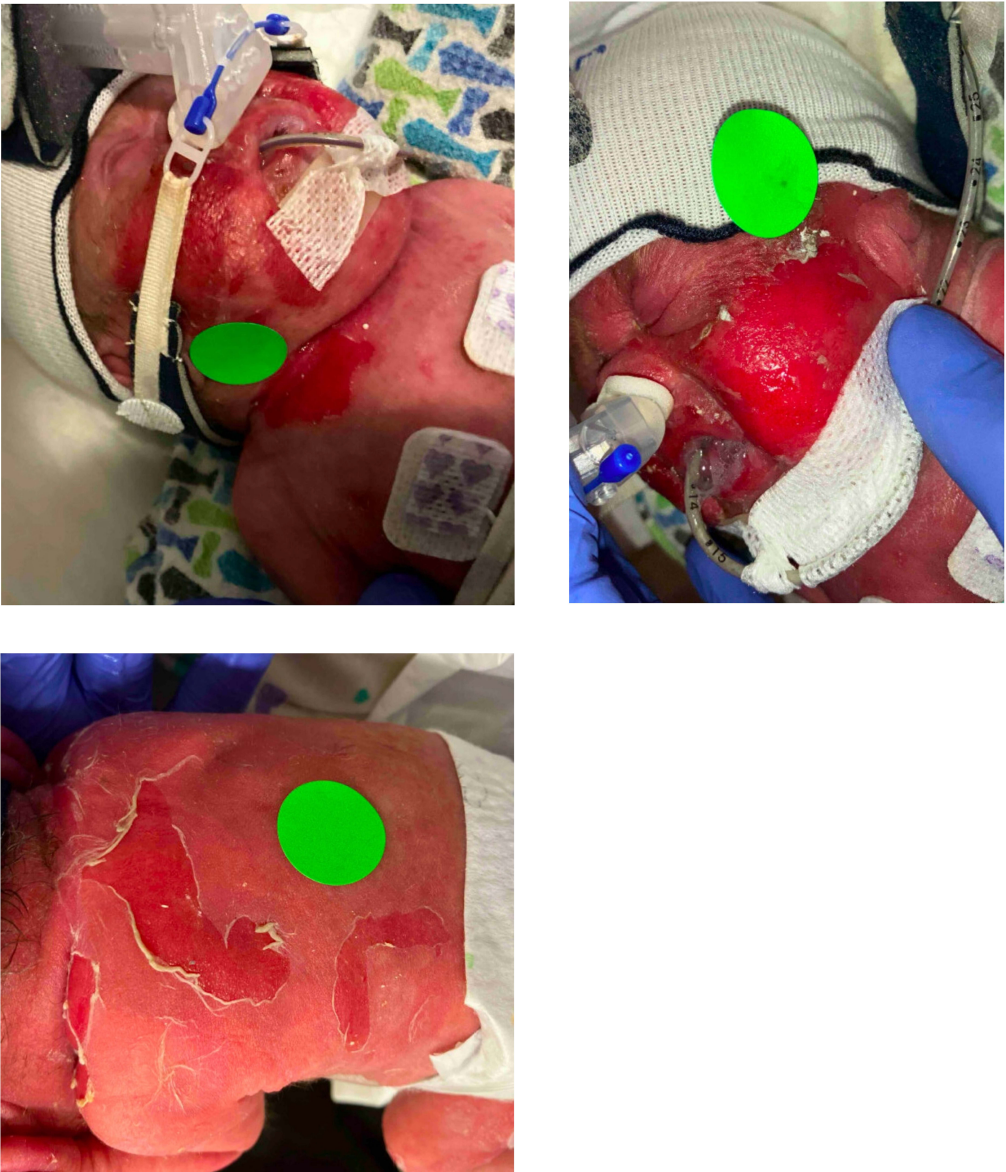
Clinical presentation at the first episode of staphylococcal scalded skin syndrome.

At about 6 and one-half weeks of life, the baby was extubated to non-invasive intermittent positive pressure ventilation via Fisher & Paykel nasal prongs/mask after a 3-week course of dexamethasone and then transitioned back to physiologic hydrocortisone. Twenty-four hours after extubation, the patient became febrile to 39.8°C and required urgent re-intubation due to apnea and desaturation events. A blood culture was obtained, and his CRP was 3.1 mg/dl. During this time, the bedside nurses noted new excoriations of areas of previously healing skin lesions (Figure 1). Nystatin powder was reordered for suspected yeast skin lesions to his neck and axilla. This excoriating and peeling skin rash quickly spread to his bilateral cheeks, chest, back, and extremities. He became acutely hypotensive, requiring fluid resuscitation and pressor support. Pediatric dermatology was consulted. Surface swabs of the open lesions were sent which were negative. A subsequent blood culture revealed the diagnosis, due to the isolation of methicillin-sensitive *Staphylococcus aureus* (MSSA)*.* Additional isolates from the blood, including *Enterococcus faecalis* and *Streptococcus epidermidis*, were believed to be true pathogens, given the patient's critical status, the presence of the Piccolo device, and the extent of skin breakdown when the blood culture was obtained.

Pediatric dermatology and infectious diseases were consulted to provide a multidisciplinary care team to develop a management care plan for this infant. With a high suspicion for SSSS, this patient received 2 g of intravenous immunoglobulins (IVIG) per dose q24 h for two doses. He was initially treated with vancomycin, cefepime, and clindamycin due to the development of septic shock until bacterial sensitivities were determined. The initial positive blood culture was followed at 24 and 48 h, with negative blood cultures after initiating antibiotic therapy. His antibiotics were changed to cefazolin for a total of 4 weeks (due to placement of the Piccolo device), vancomycin X 14 days (due to *S. epidermidis*), and clindamycin X 72 h (to reduce exotoxin production from MSSA). Vaseline was applied to denuded, erythematous, and scaling skin areas twice daily. Because he required endotracheal intubation and mechanical ventilation, a pediatric otorhinolaryngologist was also consulted who sutured his endotracheal tube to the lower gum line for securement. Adhesive remover spray (i.e., Adapt) was used if adhesives could not be avoided (i.e., endotracheal tube taping or peripheral IV or central line care). His clinical team also completed strict monitoring of his fluid losses, serum electrolytes, and temperature regulation in his isolette.

An immunodeficiency consultation was obtained for this infant, given the history of many clinical infections documented during this patient's hospital course and the family's history. The parents reported the following medical conditions:

1. The mother reported a history of Crohn's disease and preterm labor with all pregnancies.
2. A patient sibling, previously born at 25 weeks of gestation, suffered from recurrent ear infections, bronchopulmonary dysplasia, asthma, and recurrent pneumonia with hospitalizations about once per year.
3. A sibling born at 22 weeks of gestation with late-onset group B *Streptococcus*. This sibling succumbed to this infection and is deceased.
4. A sibling born at 29 weeks of gestation with a history of *Staphylococcus* scalded-skin syndrome at 1 month of age.
5. The patient's father had a sibling who died from complications relating to a *Staphylococcus* infection at 24 years of age.

Whole genome sequencing was sent on the patient and his parents, with no variants detected. Analysis of his white blood cells showed a normal distribution of neutrophils, monocytes, eosinophils, and lymphocytes. Lymphocyte subsets suggested low T-cell production for age, which improved over time and was expected to normalize as he ages. Additional immunologic workup showed normal mitogen and oxidative burst activity and normal immunoglobulin levels. However, this finding could have been modified by the IVIG infusion 1 week before the laboratory test was completed. A toll-like receptor (TLR) test was also completed to assess the function of toll-like receptors on his immune cells and was found to be within normal limits.

**Figure 2:**
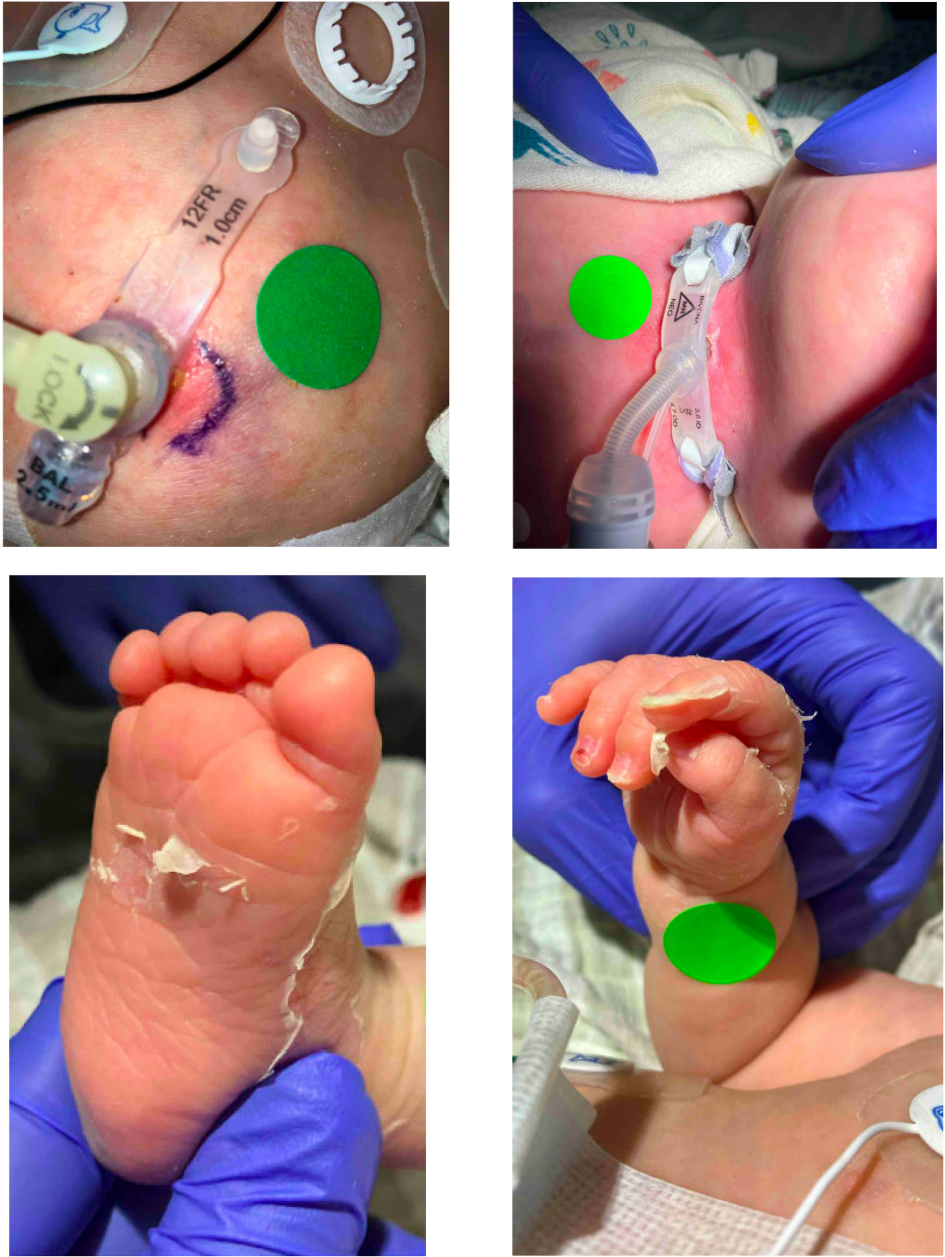
Recurrence of staphylococcal scalded skin syndrome 4 months later.

The infant went on to have a *Klebsiella oxytoca* urinary tract infection (UTI) (>100,000 colony-forming units) and was treated for 21 days with ceftriaxone per pediatric infectious disease recommendations. While he was near the end of his treatment for his UTI, he underwent tracheostomy placement around 39 weeks’ corrected gestational age due to the severity of his bronchopulmonary dysplasia, after several failed extubation attempts and multiple courses of steroid therapy. A gastrostomy tube and circumcision were completed at 42 weeks. He received routine empiric antibiotics for each of those surgeries. Five days after gastrostomy tube placement, he experienced clinical decompensation with hypercarbia and new right upper lobe opacities on the chest radiograph. An infectious diseases evaluation was completed and he was started on vancomycin and cefepime. An immunology subspecialist was reconsulted and agreed with repeat administration of IVIG at 1 g/kg/dose q24 h for two doses in the setting of a new clinical suspicion for infection. The tracheostomy aspirate resulted in 2+ gram-positive organisms that speciated to MSSA. His antibiotic coverage was narrowed to cefazolin for a recommended 10-day treatment course for pneumonia. The following day he had a new appearance of skin sloughing of his hands to the distal fingers and feet extending to the toes (Figure 2). Pediatric dermatology and infectious disease consultants were re-engaged. However, on Day 5 of treatment, he had an episode of hematochezia without pneumatosis, and his antibiotic regimen was broadened to include cefepime and metronidazole for 48 h. A BioFire gastric film array was obtained and was negative. He experienced no further episodes of hematochezia, so his antibiotic coverage was changed to cephalexin and he completed a total 10-day antibiotic course. Skin sloughing improved by Day 2 of the illness and resolved prior to spreading beyond his hands and feet. At 5 months of age, the infant was transferred to an out-of-state medical facility that was closer to his parents’ home residence. At this time, he was stable on moderate chronic lung disease ventilator settings via his tracheostomy tube and his growth was maintained around the 10th percentile for age.

## Discussion

Staphylococcal scalded skin syndrome (SSSS) is a condition that primarily affects infants and children aged 6 months to 5 years. Early diagnosis, based on characteristic dermatologic findings on physical exam, is crucial for effective management (2). Affected neonates present with fever, intense generalized erythema, and skin tenderness, with the development of superficial blisters, blister rupture, and skin denuding within 24 h of disease onset. The skin subsequently peels off in sheets, leaving a beefy red, moist, painful lesion (6). The *Nikolsky's sign* may help differentiate SSSS from other skin lesions, where the application of a gentle shearing force on intact skin leads to the upper dermis to slip to expose a plane of cleavage (6). Cultures of the exfoliative lesion are usually negative, as this injury is caused by direct disruption of desmoglein 1, a desmosomal cadherin protein that mediates cell-to-cell keratinocyte adhesion within the granular layer of the skin without epidermal necrosis and with very few inflammatory cells (3, 6–8), by hematogenous dissemination of staphylococcal exfoliative toxins (ET) A or B produced by *Staphylococcus aureus* (1, 2). In this condition, the mucosal membranes are spared because these tissues do not contain desmoglein 1 (1). The diagnosis is confirmed by isolating *S. aureus* from skin cultures, including the umbilicus, nasopharynx, conjunctiva, and diaper area, and rarely from blood cultures (<15%) (1, 6). While infants can become critically ill at presentation, this infection has an overall low associated mortality (3, 5). Differential diagnoses may also include toxic epidermal necrolysis, bullous impetigo, pemphigus, and epidermolysis bullosa (6, 9, 10).

Effective management of staphylococcal scalded skin syndrome (SSSS) requires coordinated care between pediatricians, infectious diseases specialists, and dermatologists. Even though all strains of *S. aureus* can produce toxins, only a small number (5%) release ETA and ETB and are most often associated with phage Group II, types 3A, 3B, 3C, 55, and 71, but occasionally to Groups I and III (1, 6, 11). The incubation period from skin infection to disease manifestation is between 1 and 10 days (1). Infants and young children are primarily affected due to low circulating levels of protective anti-ETA and anti-ETB antibodies. Preterm infants, especially those born at the youngest gestational ages, are exceptionally high risk due to the absence or reduced transplacental transfer of maternal IgG antibodies, which occurs in the last trimester of pregnancy (2), and reduced renal clearance of these toxins (7). *S. aureus* infection or colonization by parents, nursing, and medical staff has been a significant source of outbreaks of SSSS in neonatal intensive care units (6). Notably, nearly one-third of neonates admitted to the NICU may be colonized by *S. aureus* within 1 week of birth, but between 60% and 90% may be colonized before hospital discharge (1). Although methicillin-susceptible *S. aureus* is the causative organism in most SSSS cases, drugs effective against methicillin-resistant *S. aureus* (MRSA) should be considered in high-prevalence hospital settings, including vancomycin, linezolid, or other drugs effective against MRSA (1). Within the NICU, an estimated 7% of infants in the United States will become colonized with MRSA, and 2% of these infants will develop MRSA-mediated infection (1). Alarmingly, neonates who become MRSA-colonized during their hospitalization have a 10-fold increased risk of developing MRSA bacteremia (1). Clindamycin, a ribosomal inhibitor, is commonly recommended as a first-line agent in SSSS because it has been found to inhibit toxin production. However, most isolates are resistant to this drug. Previous studies suggest a lack of benefit between patients treated with and without clindamycin (5). Antibiotics should be administered in coordination with a pediatric infectious diseases specialist and guided by culture-associated *S. aureus* identification and sensitivity data (9). Consultation by dermatology may also be beneficial to maximize therapies relating to wound management and healing.

Infants with SSSS should be placed in an isolette air-controlled bed. Radiant heat warmers should be avoided because of associated contraindications with petroleum-based products and their drying nature, which may impair wound healing (9). The strict enforcement of good hand hygiene practices is not just a recommendation but a responsibility that all care providers must uphold to prevent the spread of this bacterium within the NICU (9, 12). Moreover, the denuded skin lesions are at an increased risk for secondary bacterial infections and excessive water loss, similar to those experienced by burn patients. Clinical management also involves wound cleansing, moist dressings, and the application of antiseptic or hydrocolloid ointment and creams. The application of Aquaphor or Vaseline to the affected skin can reduce fluid loss and protect exposed areas (10). In very preterm infants, the use of saturated gauze may cause hypothermia or may stick to the infant's skin when it begins to dry (9). In these infants, the use of Mepitel, a silicone-based dressing that is tacky on only one side, has been used with some success (9, 10). The use of IVIG has been used to acutely increase circulating anti-ETA and anti-ETB antibodies, such as in the case presented here. Commercially available IVIG is refined from the sera of 10,000–20,000 individuals and contains high levels of anti-ETA antibodies (2). Pain medications should be considered if clinically indicated, especially during dressing changes. Non-steroidal anti-inflammatory drugs (NSAIDs) are not recommended as they can inhibit the renal clearance of ET and attenuate host-immune cytokine pathways (12).

Recurrent staphylococcal scaled skin infections are rare, with the majority of case reports occurring in low birthweight neonates and the time between initial onset and recurrence of <30 days (13–15). This case is unique in that the infant had 3 months between the initial onset and recurrence with marked improvement in symptoms with early administration of IVIG and prompt treatment of his MSSA infection. Although our current testing capabilities did not detect a specific immune deficiency unrelated to prematurity, it cannot be completely ruled out, given the possible contributory family history. At a minimum, inherent deficiencies of innate and adaptive immune responses exhibited by preterm infants, especially those extremely preterm, place them at exceptionally high risk for infectious diseases and underscore the benefits of multidisciplinary subspecialty care in clinical management.

## Data Availability

The original contributions presented in the study are included in the article/Supplementary Material, further inquiries can be directed to the corresponding author.
